# Joint Clinical Assessment in the EU HTA Regulation—Would Drugs Supported by Single-Arm Trials Fit Under Evaluation?

**DOI:** 10.3390/jmahp14020036

**Published:** 2026-06-22

**Authors:** Krzysztof Kloc, Mondher Toumi, Elżbieta Łukomska, Malwina Kowalska, Inez Tyrała-Chowaniec, Steven Simoens, Jürgen Wasem, Laurent Boyer, Claude Dussart, Pascal Auquier

**Affiliations:** 1 Clever-Access, 30-415 Krakow, Poland; 2 Public Health Department, Université d’Aix-Marseille, 13385 Marseille, France; mondher.toumi@univ-amu.fr (M.T.);; 3 Department of Pharmaceutical and Pharmacological Sciences, Katholieke Universiteit Leuven, 3000 Leuven, Belgium; 4 Department of Health Care Management, Universität Duisburg-Essen, 45127 Essen, Germany; 5 Health, Systemic, Process (P2S) Research Unit (UR 4129), Université Claude Bernard, 69372 Lyon, France

**Keywords:** joint clinical assessment, single-arm trial, health technology assessment

## Abstract

The Joint Clinical Assessment (JCA) evaluates the relative effectiveness (RE) of interventions over comparators. While randomised control trials (RCTs) are considered the gold standard, single-arm trials (SATs) require an external control for accurate RE estimation. This study reviewed Health Technology Assessment (HTA) outcomes for medicinal products supported by SATs in France, Germany, Poland, and Spain, and simulated the JCA for these products based on evidence submitted in France. Among HTA evaluations published in France in 2019–2024, 16% were SAT-driven, and 5.6% of them included external controls. SAT-supported drugs had a high reimbursement approval rate (74%) and showed better HTA outcomes when controls were used. In Germany, 64% of SAT-based HTA outcomes indicated no added benefit and 30% a non-quantifiable benefit. In Poland and Spain, 63% and 72% HTA evaluations recommend reimbursement, respectively. Despite wide acceptance by Member States, experts determined that 94% of SAT-supported products would not qualify for JCA review due to insufficient evidence. Only 6% would qualify for JCA for a likely limited number of PICOs (Population–Intervention–Comparator–Outcome), but the certainty rating would be low. These findings suggest that SATs, as primary evidence, may not be suitable for JCA, potentially undermining HTA in EU Member States.

## 1. Introduction

The implementation of the Joint Clinical Assessment (JCA) in the European Union (EU) [1] represents a significant reform in how new health technologies, particularly medicinal products, are evaluated across Member States (MS). This initiative is part of the broader Health Technology Assessment (HTA) Regulation (EU 2021/2282) [2], which aims to streamline processes and enhance patient access to innovative treatments [1,2].

The HTA Regulation was enacted in January 2022, laying the groundwork for the JCA framework. It aims to standardise assessments across the EU, reducing redundancy and improving access to health technologies [1,3]. Starting from 12 January 2025, the JCA applies to new medicines intended for cancer treatment and advanced therapy medicinal products (ATMPs), expanding to a broader inclusion in 2028 to orphan drugs, and to all other medicinal products by 2030 as foreseen in the regulation [3,4].

The objective of assessments under JCA is to support national HTA processes with a scientific analysis of clinical evidence conducted in a standardised and harmonised manner [3]. The primary output will be a detailed report generated by assessors and co-assessors based on the review of submitted evidence from health technology developers (HTDs). This report will summarise clinical evidence, focusing on the relative effectiveness and safety without making judgments about clinical added value or cost-effectiveness [1]. The report is intended to provide MS with essential data to inform their own national assessments and decision-making processes regarding the adoption of new health technology. MS are required to provide due consideration to these reports but retain the responsibility for making their own conclusions regarding clinical added benefit, value, and reimbursement decisions based on their national HTA framework and health priorities [5].

The JCA utilises a standardised methodology developed through previous collaborative efforts by EUnetHTA, which concluded its work in September 2023. The assessment is driven by a set of procedural guidance documents, methodological and practical guidelines, and template documents, which have been successively released since March 2024 [6]. The PICO (Population, Intervention, Comparator, Outcome) framework is adopted to define the scope of the assessment and ensure that the evaluations are relevant to specific patient populations and treatment contexts [5].

The comparator is an essential component of the assessment because JCA focuses on assessing the degree of certainty and the size of the relative effectiveness and safety of a health technology compared to existing treatments or standard care. HTDs are expected to provide robust data demonstrating how their product compares to existing treatments under each defined PICO. Well-designed randomised clinical trials (RCTs) with a low risk of bias are considered the “gold standard” for informing estimates of treatment relative effectiveness, and direct comparisons based on adequate RCTs should be applied whenever possible. If RCTs are not feasible, single-arm trials (SATs) may be accepted, especially when combined with an external control arm [7].

The validity of clinical studies used for the JCA is determined by three key dimensions: internal validity, external validity, and statistical precision. A JCA report should explicitly state the study design used and provide a comprehensive assessment of the certainty of the reported relative effectiveness, taking into account all three dimensions [8]. Statistical precision is typically communicated using confidence intervals (CIs), while external validity assessment is more complex and depends on how well the study design resembles the real-life target population and treatment conditions under the various PICOs. To assess the risk of bias associated with the internal validity of RCTs, the Cochrane Risk of Bias (RoB) tool, specifically the RoB version 1, is recommended [8].

In the case of SATs, no formal rules for assessing the risk of bias of the SAT are considered because the lack of a control group precludes performing direct relative effectiveness assessments. However, when used with an external source of data as a control arm, the certainty of the relative effectiveness is determined by the internal validity, external validity and statistical precision of the outcome of indirect treatment comparison (ITC), rather than the single-arm trial by itself [8].

Single-arm trials are utilised primarily in specific contexts where traditional RCTs may not be feasible or ethical. Most often, these are rare diseases, including rare cancers, where the patient population is too small to achieve the statistical power required in RCTs. Also, in life-threatening conditions where standard care is not effective, preventing patients in the comparator arm from receiving potentially effective treatment may be considered unethical [9].

SATs are an important tool in clinical research, contributing to regulatory decision-making and ultimately improving patient access to innovative therapies [10].

The objective of the research was to compare the current acceptability of medicinal products evaluated based on SAT by the selected national HTA agencies with simulated probable outcomes of the assessment of the same products under the JCA framework, and to anticipate potential implications for the future market access of such products after adopting JCA in the EU.

## 2. Materials and Methods

Medicinal products which were assessed based on SAT were identified using detailed information about HTA processes in France collected in the NaviHTA database (https://www.inovintell.com/ (accessed on 31 March 2025)) (NaviHTA is a database of Inovintell company, Kraków, Poland, containing comprehensive information extracted from HTA reports of key European HTA agencies.) Database records referring to HTA reports issued by the French HTA agency (Haute Autorité de Santé; HAS) between 2019 and 2024 were retrieved and screened for HTA processes in which SATs were the primary source of clinical efficacy evidence. Reports based on direct comparative clinical trials, including crossover studies, were excluded. Where a report contained two or more patient subgroups with distinct assessments, these were treated as separate HTA processes. The assessments were analysed in terms of the indication, presence of unanchored ITC (For clarity, ITC is used here as an umbrella term. Subtypes include unanchored ITCs with external controls and statistical approaches such as MAIC. In brief, ITCs allow comparisons between treatments when no head-to-head trial exists, with MAIC adopting a statistical method to adjusts for differences in patient characteristics between studies.) with external control, the use of a matching-adjusted indirect comparison (MAIC), and the final rating of actual benefit (Service Médical Rendu, SMR) and improvement in the actual benefit (Amélioration du Service Médical Rendu, ASMR). Since the ITC could be available only for selected subpopulations within the same HTA report and, ultimately, drive different HTA outcomes, the availability of ITCs was reported for individual HTA processes. The analysis adopts France as the anchor country: SAT-supported products were identified through the French HAS/NaviHTA database, and the same products were then traced through the HTA databases of Germany, Poland, and Spain (see below). By construction, SAT-supported products not reviewed by HAS during the study period—but potentially reviewed in other countries—are not captured in this analysis. The French set therefore constitutes both the reference universe and the structural upper bound for the cross-country comparison; other countries can, at most, equal but cannot exceed the French count by design.

For all products identified in France as having HTA driven by SAT, corresponding assessments were searched on the websites of the German Federal Joint Committee (Gemeinsamer Bundesausschuss, G-BA), the Agency for Health Technology Assessment and Tariff System in Poland (Agencja Oceny Technologii Medycznych i Taryfikacji, AOTMiT), and the Spanish Agency of Medicines and Medical Devices (Agencia Española de Medicamentos y Productos Sanitarios, AEMPS), in collaboration with the Network of Health Technology Assessment Agencies (Red de Agencias de Evaluación de Tecnologías Sanitarias y Prestaciones del SNS, REDETS). HTA processes identified in these countries were analysed based on the same criteria as in France regarding the use of direct comparative data or ITC, and for different assessment outcomes for subgroups, to determine individual HTA evaluations. For each assessment, data were extracted on the final outcome, including the added benefit rating and resolution statement in Germany, the reimbursement recommendation and type of market access pathway in Poland, and the assessment outcome and conclusion statement in Spain. Specifically for Germany, since the reimbursement is systematic for all newly approved products, and the HTA outcomes do not directly determine the reimbursement status, the availability of products on the national market was extracted from the global pricing database and considered as a proxy of reimbursement to allow comparison with other jurisdictions [11].

Because SAT data could be more acceptable in rare indications, where recruitment is challenging, as well as due to a simplified HTA process in Germany of orphan products, the orphan status of all products included in the analysis was identified on the European Medicines Agency (EMA) database [12] to provide additional decisional context for the products.

The JCA simulation for products with SAT was conducted based on their clinical data used for HTA in France, extracted from the NaviHTA database. When no ITC was available, products were considered as not assessable in line with JCA Guidance on Validity of Clinical Studies [8], following the statement that uncontrolled trials, when they are the only source of data submitted as evidence, do not allow relative effectiveness assessment. When ITC was available, a JCA was performed. Guidance on Validity of Clinical Studies [8] and Methodological Guideline for Quantitative Evidence Synthesis: Direct and Indirect Comparisons [7] were applied independently by two experts in the field of market access and HTA in Europe to assess the evidence of a product and anticipate its probable acceptability under the JCA process. The outcomes of their assessment were reported for each product and compared. In the case of disagreement, direct discussion and reconciliation would have been conducted with an attempt to reach consensus. If disagreement persisted, a third independent expert of the same profile would have been appointed to arbitrate between the two initial assessments. In practice, no disagreements required escalation to a third reviewer.

Acceptability was defined in France, Poland, and Spain as achieving reimbursement for a single product for at least one of the indications or subgroups that was filed to HTA bodies. In Germany, products are reimbursed once the price is accepted by HTDs, and HTA informs the price rather than the reimbursement. Therefore, acceptability was defined as market availability and reimbursement.

For JCA, acceptability was defined as reaching a level of certainty for the comparative effectiveness outcome that surpassed the lowest acceptable level. The acceptability derived from the JCA simulation was then compared to the acceptability results from the four countries.

The HTA documents were reviewed mostly in English. French assessments were accessed in English through the NaviHTA database. German decisions, when available in English, were reviewed in that language; otherwise, translated versions were analysed. Spanish reports were analysed following translation into English. Polish recommendations were reviewed in the native Polish language.

## 3. Results

### 3.1. Characteristics of Products with SAT

At the time of the analysis (January 2025), the NaviHTA database contained 883 HTA outcomes in France issued between 1 January 2019 and 31 December 2024. Among those, 141 assessments (16%) were based on an evidence package containing an SAT as the key source of clinical evidence. As a single HTA report could contain more than one HTA evaluation, for example, for subgroups, 141 assessments come from 116 HTA reports for 96 different medicinal products.

Nearly half of the products were for oncological indications (44%, 42 products), of which 48% (20 products) were for haematological cancers. According to the EMA database, 33% of products (31) had an orphan drug status, of which half of them (15) were oncology products [13]. The most prevalent oncology indications were lymphomas (10 products), followed by non-small cell lung cancer (7 products) and multiple myeloma (6 products). Among non-oncology products, the most frequent indications were human immunodeficiency virus (HIV) infection (seven products), chronic hepatitis C (four products), cystic fibrosis and haemophilia A (three products each). Ten drugs (10%) were classified by the EMA as ATMPs [13].

Out of all 141 HTA evaluations driven by SAT, only 8 assessments (6%, 6 products) included any ITC with an external control arm to allow assessment of relative effectiveness. Among those, four assessments (three products) were supported by ITC, which adopted the MAIC approach, and the remaining four assessments (three products) were based on unadjusted methodology.

### 3.2. Analysis of HTA Outcomes in Selected EU Countries

#### 3.2.1. France

**Figure 1:**
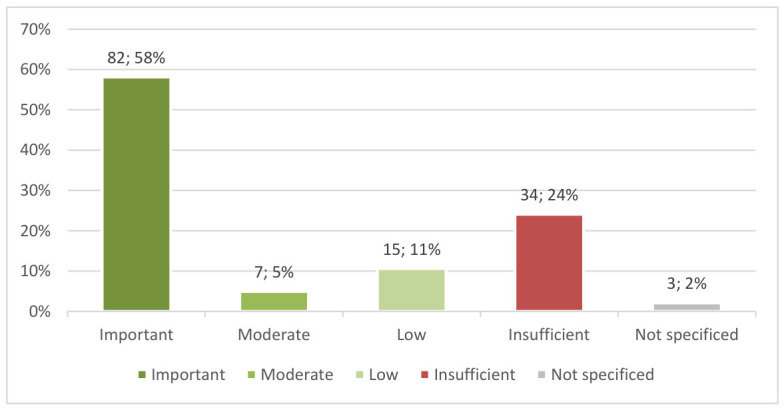
Actual benefit (SMR) ratings in the HTA outcomes based on SAT in France.

Out of all 141 HTA evaluations for the total of 96 different products, a significant majority (74%, 104 assessments) was positive, with actual benefit (SMR) ratings ranging from low to important. Positive assessments concerned 82 different products. Over half of the HTA outcomes (58%, 82 assessments) indicated an important SMR level, as illustrated in Figure 1.

Eight HTA processes for six products included an ITC, and all indicated an important SMR, except one. However, the negative HTA outcome concerned one subgroup of patients with neuromyelitis optica spectrum disorder treated with Ultomiris^®^ (ravulizumab), while this product received a positive SMR in another subgroup with this indication (patients who failed background immunosuppressive therapy).

**Figure 2:**
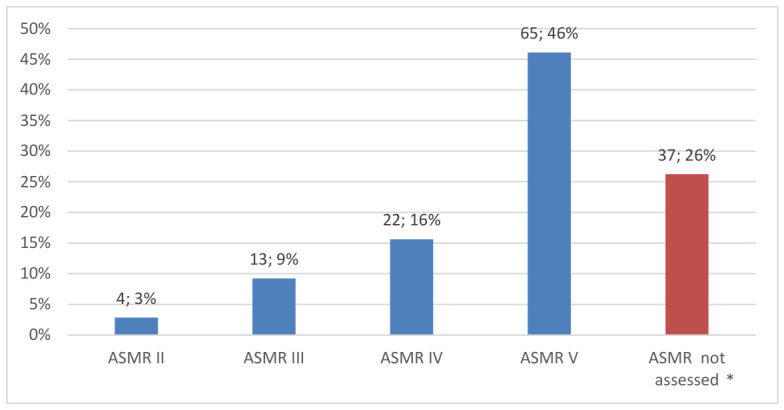
Improvement in actual benefit (ASMR) ratings in the HTA outcomes based on SAT in France. * ASMR was not assessed due to insufficient SMR or not specified.

Regarding the assessment of improvement in actual benefit (ASMR), 46% of HTA outcomes (65 assessments) indicated no improvement (ASMR V), and the frequency of assessments with improved ASMR decreased with the decreasing level of ASMR (16%, 9% and 3% for ASMR IV, III and II, respectively), as depicted in Figure 2.

**Figure 3:**
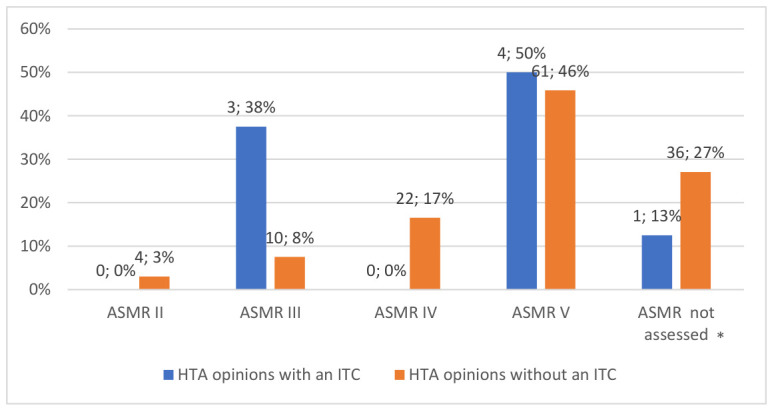
Improvement in actual benefit (ASMR) ratings in the HTA outcomes, depending on the use of an ITC. * ASMR was not assessed due to insufficient SMR or not specified.

Assessments supported by an ITC obtained higher ratings of ASMR: the rate of assessments with ASMR III was much higher when supported by an ITC compared to the assessments without external control (38% and 8%, respectively). The rate of assessments with ASMR V was similar in both groups. These patterns are summarised in Figure 3. However, the number of HTA evaluations with an ITC was low (eight assessments for six products).

#### 3.2.2. Germany

**Figure 4:**
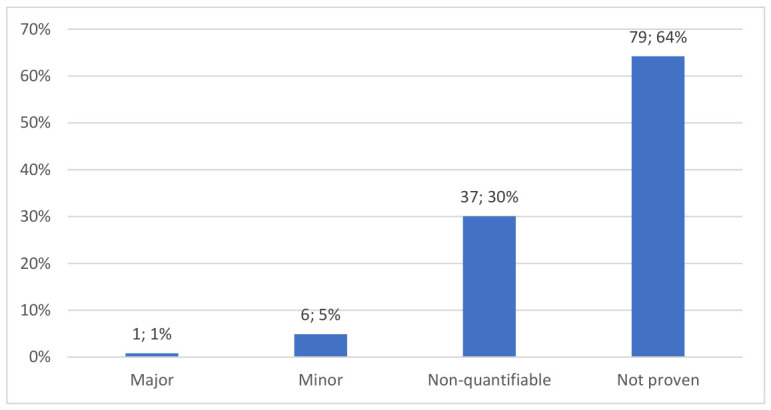
Added benefit ratings in the HTA outcomes in Germany.

The search for HTA evaluations for products identified as having SAT in France revealed 123 assessments in Germany for a total of 65 medicinal products. A large majority of the HTA outcomes (64%, 79 assessments) were rated as no proof of added benefit. For most of the remaining cases (30%, 37 assessments), HTA evaluations were rated as having non-quantifiable added benefits. It should be noted that in the German AMNOG (Act on the Reform of the Market for Medicinal Products, Arzneimittelmarktneuordnungsgesetz) framework, “non-quantifiable added benefit” does not represent a benefit category lower than “minor”; rather, it designates a plausible but imprecisely quantifiable therapeutic gain, which may correspond to minor, considerable, or even major benefit levels and is frequently regarded as a favourable outcome in price negotiations. This category is retained as a distinct analytical unit in this study because the inability to quantify the magnitude of comparative benefit is directly relevant to assessing the evidentiary sufficiency of SAT-based submissions—a consideration central to the JCA simulation performed herein. The defining feature of the AMNOG “non-quantifiable” rating—an evidence package judged plausible enough to indicate that an added benefit exists, but methodologically insufficient to allow a structured quantification of the magnitude of comparative effect—maps directly onto the analytical situation the JCA framework encounters with SAT-supported products under the Guidance on Validity of Clinical Studies and the Methodological Guideline for Quantitative Evidence Synthesis. Aggregating this outcome with the quantifiable benefit categories would obscure precisely the analytical signal central to our research question, namely that the German system has historically accepted descriptive but unquantifiable evidence as a basis for added benefit ratings—a flexibility that the harmonised JCA framework is structurally designed not to replicate. In 6% of the HTA outcomes (seven assessments), despite SAT-derived clinical data, it was possible to quantify the level of added benefit. One assessment indicated a major added benefit, while the remaining six were rated as having minor added benefit, as shown in Figure 4. Those HTA outcomes concerned three products: Brineura^®^ (cerliponase alfa), Libtayo^®^ (cemiplimab) and Sovaldi^®^ (sofosbuvir), where the latter was assessed separately in five different subgroups of patients with hepatitis C, all of which received an added benefit.

Considering the high requirement for direct comparative data in Germany, seven assessments, which were based on SAT and included added benefit recognition, were reviewed for justification.

In the justification of the major added benefit rating for Brineura^®^ (BioMarin International Limited, San Rafael, CA, USA), the G-BA noted uncertainties associated with the historical comparisons but indicated the rarity of the disease, the paediatric patient population, and the deterministic disease course. The assessment also considered long-term data confirming previous findings of morbidity and mortality advantages [14]. In the HTA evaluation of Libtayo^®^ (Regeneron Pharmaceuticals, New York, NY, USA and Sanofi, Paris, France), the minor added benefit assessment was justified based on the lack of relevant effects on clinical response under the comparator therapy of best supportive care, while for the assessed treatment, a hint of advantage in clinical response was found [15]. The justification for the minor added benefit assessment of Sovaldi^®^ (Gilead Sciences, Inc., Foster City, CA, USA) in all five subgroups of patients with chronic hepatitis C indicated that, despite not meeting the highest evidence standards typically required, single-arm studies compared to historical controls are acceptable because the disease can cause severe complications, and current interferon-based treatments are lengthy and have serious side effects [16].

Among all 65 medicinal products assessed in Germany, four products have been withdrawn, giving an eligibility rate of 94%. HTA evaluations for two of them indicated no added benefit (Gavreto^®^ (Rigel Pharmaceuticals, San Francisco, CA, USA) and Tabrecta^®^ (Novartis Pharmaceuticals Corporation, East Hanover, NJ, USA), while the other two products were assigned a non-quantifiable added benefit (Copiktra^®^ (Secura Bio Limited, Dublin, Ireland) and Zynteglo^®^ (Genetix Biotherapeutics, Somerville, MA, USA). Publicly available information confirms that the withdrawal of Tabrecta^®^ was directly associated with a negative added benefit assessment [17]. In the case of Zynteglo^®^, despite the non-quantifiable added benefit, it was withdrawn after failing to reach an agreement with health authorities on the treatment’s price [18]. Gavreto^®^ was withdrawn from the whole EU in January 2025 due to commercial reasons [19]. Copiktra^®^ is still available in the EU, but according to the pricing database, it was withdrawn from Germany a few months before the publication of the G-BA resolution in July 2022.

#### 3.2.3. Poland

**Figure 5:**
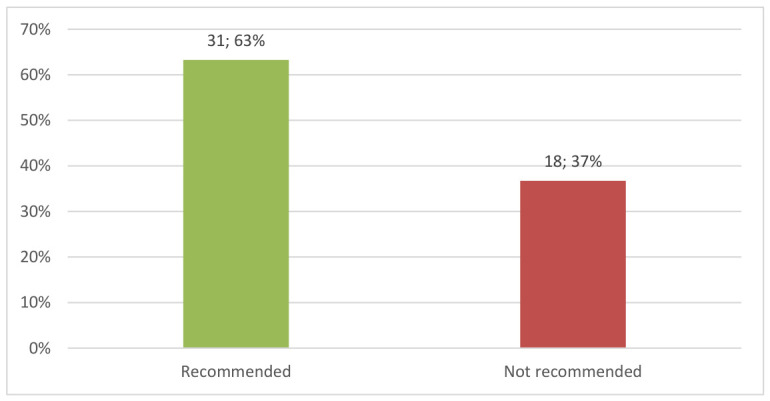
Reimbursement recommendations in the HTA outcomes in Poland.

Among 96 products identified as having SAT in France, only 40 had HTA evaluations in Poland, with 49 HTA outcomes available. A majority of the HTA evaluations (63%, 31 assessments) were positive and recommended reimbursement of the products, which were evaluated based on SATs, as shown in Figure 5.

**Figure 6:**
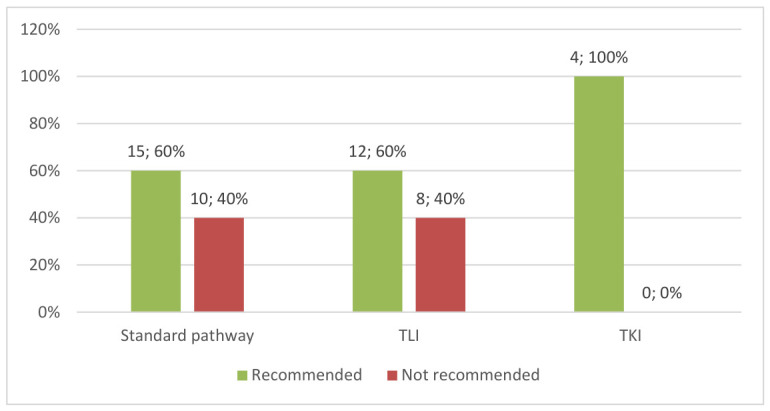
Reimbursement recommendations in the HTA outcomes in Poland, depending on the market access pathway.

Since different market access pathways could be applied in Poland depending on the product profile (a standard pathway, a pathway for drug technologies with a high level of innovation (TLI), and a pathway for drug technologies with high clinical value (TLK) [20,21]), HTA outcomes were analysed separately for each pathway. Twenty-two products were assessed using the standard pathway, 18 were assessed using TLI, and 2 were assessed using TLK pathways. As shown in Figure 6, no significant differences were observed between the pathways, with positive and negative assessment rates consistent with the overall analysis. The standard pathway and the TLI showed 60% of HTA evaluations being positive, concerning 13 and 10 products, respectively. The TLK pathway seemed to allow for higher acceptability than other pathways, but since the rate was based only on four assessments for two products, the trend is highly uncertain.

#### 3.2.4. Spain

**Figure 7:**
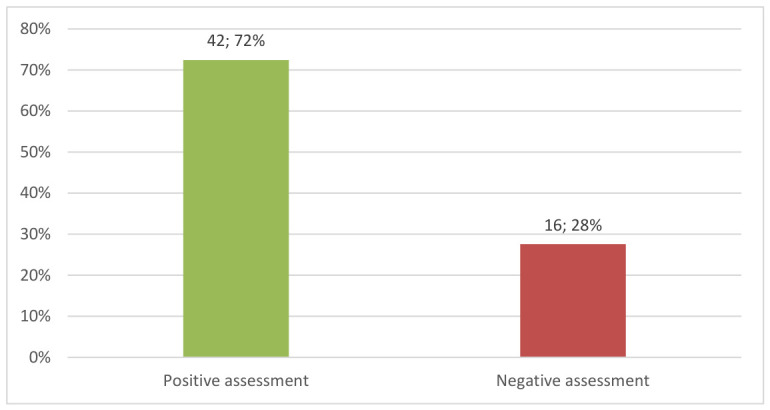
HTA outcomes in Spain.

Analysis of the AEMPS website revealed 58 HTA outcomes for 46 products with SAT. The majority of the HTA evaluations (72%, 48 assessments) were positive, while the remaining assessments (28%, 16 assessments) were negative, as presented in Figure 7.

Many drugs were conditionally accepted based on limited data, pending further research and long-term efficacy and safety results to establish precise positioning. The conclusions of the HTA agency also indicated specific patient populations characterised by the high severity of the disease or lack of alternatives and suggested individual risk/benefit assessments when making treatment decisions.

### 3.3. Simulation of JCA of Drugs Supported by SATs and Comparison with National HTA Outcomes

Analysis of HTA evaluations for medicinal products with SAT in countries of scope showed that, despite limitations of the single-arm design, the majority of products have been accepted at the HTA stage (Table 1).

The JCA simulation did not show a discrepancy between experts. Neither conciliation nor arbitration was requested.

All products with no indirect comparative evidence (94%, 90 products) have been classified as non-assessable and excluded from the assessment. For such products, no JCA report would be issued.

Six products with an ITC have been assessed under JCA guidance, which represents 6% of all products evaluated in France based on SAT. These products were considered qualified for JCA for a likely limited number of PICOs (Population-Intervention-Comparator-Outcome). However, in line with the JCA guidance documents, all of them received the lowest degree of certainty because of unanchored ITC.

The comparison between JCA and countries in scope is reported in Table 1.

## 4. Discussion

This study aimed to assess the proportion of SAT-supported products that gain reimbursement or market access availability in France, Germany, Spain, and Poland and compare it with the anticipated acceptability of these products in the case of JCA. JCA outcomes were generated through a simulation performed by two experts in the field. The selected countries primarily rely on direct comparative evidence for granting availability, but this study confirms that SAT-supported products have a high availability rate in those countries, despite not meeting comparative evidence requirements. In contrast, the simulated JCA-related availability was zero. Although the JCA report does not recommend reimbursement per se, Article 9 of the EU HTA regulation specifically requires that the JCA report provide a degree of certainty of the relative effectiveness of the evaluated product. Article 13 indicates that MS are required to give due consideration to the JCA report and document how they did it in the appraisal process. The Regulation itself explicitly contemplates that JCA outputs inform pricing and reimbursement decisions, while not predetermining them. Recital 5 of Regulation (EU) 2021/2282 acknowledges that the outcome of HTA is “used to inform decisions concerning the allocation of budgetary resources in the field of health, for example in relation to establishing the pricing or reimbursement levels of health technologies”; Recital 14 stipulates that JCA outcomes should not “predetermine subsequent decisions on pricing and reimbursement”, a prohibition that presupposes the factual capacity to do so; and Recital 36 indicates that joint clinical assessments should “effectively facilitate market access and contribute to the timely availability of innovative health technologies for patients”. The JCA report does not therefore contain a value judgement; rather, it provides a scientifically grounded conclusion on the degree of certainty of relative effects that is, by the explicit design of the Regulation, intended to inform—without predetermining—national appraisal, pricing, and reimbursement processes. Member States retain the sole competence to draw value-laden conclusions on clinical added value and to take pricing and reimbursement decisions (Recitals 26 and 31; Article 1(2)). Our analysis is therefore concerned not with the JCA usurping any function reserved to Member States, but with the structurally predictable consequences of the JCA’s lawful scientific output for SAT-supported products. It should be noted that “degree of certainty” is the statutory wording of Articles 8(6) and 9(1)(b) of Regulation (EU) 2021/2282 and is used throughout this manuscript in accordance with the Regulation’s own language. Under any operationalisation of this requirement—whether descriptive or qualitative in form—JCA reports for SAT-based products will inevitably characterise the available evidence as carrying very high uncertainty, given the structural absence of a valid controlled comparator. This study highlights the difference between the current availability of SAT-supported products and the potential outcomes of JCA, which would need to be considered by MS. The implications are discussed later in this section.

SATs remain important tools for drug development. However, a reflection paper of the EMA outlines several trial aspects which should be taken into account when assessing the efficacy of medicines based on SATs. These include a selection of outcomes that cannot occur without the treatment and can be objectively measured, e.g., specific biological measures, cure or survival beyond time, which would be expected based on the natural course of the disease. In the case of continuous endpoints, a cut-off value should be predefined, which cannot be achieved without treatment. The trial population should be well-defined, and its characteristics should reflect the assumptions on the natural course of the disease. If there is knowledge about prognostic or predictive biomarkers, they should also be reflected in the SAT sample [10].

### 4.1. SAT-Supported Products Enjoy Fair Recognition

Several MS HTA frameworks require direct comparative evidence and have a low appetite for indirect comparative evidence (France and Germany). Therefore, it is notable to observe a high rate of product acceptability [22]. The rates are 85% for France, 76% for Spain, 90% for Poland, and 94% for Germany.

France has strict comparative evidence requirements for reimbursement. Despite this, many drugs receive favourable recommendations, even though parliament mandates direct comparative evidence, as well as the HAS doctrine [23]. Notably, some medicines achieved ASMR II and III classifications with just single-arm trials. In France, ITCs are considered supportive [24].

As per the German Act on the Reform of the Market for Medicinal Products (Arzneimittelmarktneuordnungsgesetz, AMNOG) regulations, the actual patient-relevant benefit of a new drug is required to be demonstrated through a comparison with an established drug or treatment strategy. In the absence of direct comparisons, indirect comparative data may be used. However, non-adjusted indirect comparisons are considered unacceptable [25].

In Germany, all orphan-designated medicinal products are legally entitled to receive an additional benefit [26]. In consequence, orphan-designated medicinal products supported by SAT are granted an additional benefit by the G-BA, often unquantifiable, even in the absence of comparative evidence [27]. This may increase the number of SAT-supported medicinal products reported as having a positive additional benefit, because without this regulation, they would not qualify for an additional benefit. Despite the stringent requirement for relative effectiveness evidence, seven SAT-based HTA processes concluded with a quantifiable additional benefit, with one indicating a major additional benefit.

In Poland, while many medicinal products are reimbursed with an SAT, only 40 out of 96 have been assessed. Even if non-assessed products are considered to have negative HTA outcomes, the reimbursement rate would remain high, especially given Poland’s lower Gross Domestic Product per inhabitant in the EU and the usual high price of such products.

In Spain, similarly to Poland, only part of the products assessed based on SAT (46 out of 96) underwent evaluation by the HTA agency. However, the rate of positive options is even higher than in Poland, indicating the significant acceptability of SAT-based products despite the associated uncertainties.

Different numbers of products with SAT were evaluated across the MS of scope. It could be explained by distinct HTA framework requirements and various funding models. The difference between France and Germany (96 and 65 products, respectively) is most plausibly explained by structural differences in HTA scope and assessment timing between the two systems. The HAS/CT database covers a broader range of product types, including additional indications and, in some cases, biosimilars or generics, and assessments often occur after market entry; the AMNOG framework focuses on early benefit assessments for new active substances and their extensions at the time of market launch, applying a narrower product scope. These structural factors are likely the primary driver of the numerical gap. Additionally, anticipatory launch behaviour by manufacturers—while not excluded as a contributing factor and supported by available pricing data—cannot be precisely quantified from the current data and should not be over-interpreted as a causal explanation. Lower numbers of products assessed in Spain and Poland (46 and 40, respectively) could result from the discouraging negotiation process following the assessment, which is highly driven by budgetary capabilities rather than the evidence and value of the product.

Our results regarding reimbursement and acceptability align with several published studies using various methodologies and scopes. Analyses of HTA recommendations in multiple countries (including Germany, the United Kingdom, Sweden, the Netherlands, Canada, and Australia) have demonstrated that SAT-based evidence for medicinal products does not necessarily result in negative outcomes. In those studies, many products supported by SATs received positive HTA recommendations at a rate comparable to those based on randomised data [28].

Recent research on HTA outcomes in France and Germany for products with SATs between January 2020 and May 2022 confirms high success rates. In France, 55% of reports (11 of 20) had positive outcomes, while in Germany, 85% (23 of 27) were considered positive [29]. In Portugal, eight out of nine HTA processes for products with SAT from January 2017 to April 2022 received positive reimbursement decisions with non-quantifiable additional benefits [30].

### 4.2. The Low Value of JCA for SAT-Supported Products Justifies Their Exemption from JCA

The guidance document on the validity of clinical studies takes a position regarding SATs. They are not considered suitable for assessing relative effectiveness and, without an indirect comparison, will be classified as not assessable and not assessed [8]. Following this position, such classification applies to 90 out of 96 products in our sample.

ITC methodologies are inherently unanchored for SAT, resulting in the lowest degree of certainty of relative effectiveness [22]. Moreover, as indicated in the JCA guidelines on quantitative evidence synthesis, unanchored indirect comparisons break randomisation and require the assumption of “conditional constancy of absolute effects” [7]. This assumption means that the absolute outcome in treatment arms is constant at any given level of prognostic variables and effect modifiers. It is usually unjustifiable in practice, making the ITC without a common comparator highly unreliable [7,31]. Based on that, it is highly probable that even if an ITC is available for some products, such evidence would be classified as highly confounded. This would likely lead to a conclusion of the lowest degree of certainty of the relative effectiveness, potentially impacting their assessability by the MS.

This raises several questions:

While most medicinal products supported by SATs get reimbursed, what impact will a JCA report concluding with a very low degree of certainty of relative effectiveness have? Since MS HTA bodies must document the due consideration of JCA reports, this could complicate reimbursement and additional benefit assessments, including major benefits or ASMR II or III.

Given that JCA for SAT-supported products will likely be inconclusive with known outcomes before filing, does the utility of JCA justify a significant effort associated with the centralised assessment and its potential implications? The JCA is a long-term process lasting more than a year and is very resource-intensive. Running such a process while the outcome is known beforehand may be considered questionable. The JCA is decontextualised, whereas the MS HTA bodies’ assessment is contextualised, integrating various constraints and a face-value benefit appreciation that can influence the appraisal. For instance, the high level of unmet needs and the burden of these conditions are part of the assessment in France, Spain, and Poland. Given this context, the added value of JCA is debatable.

Exempting such medicinal products from JCA might be beneficial and would:

- Prevent potential restrictions on access to important interventions;
- Align with current HTA practices that often favour SAT-supported products;
- Reduce the workload for the already stretched Member State Coordination Group on Health Technology Assessment (HTACG) resources.

Medicinal products supported by SAT and eligible for JCA between 2025 and 2028 are already developed or in late clinical stages. Thus, JCA guidelines (available since 2024) have come too late for consideration in their development. Exempting these products from JCA and reconsidering them in 2028 seems reasonable.

Concerns could be raised that HTDs will avoid JCA by engaging more in SAT. To assess such risk, it should be considered that 25% or more of SAT-supported products do not get reimbursement. If SATs are increasingly used instead of RCTs, the rejection rate would inevitably increase. HTA and payers would penalise inappropriate decisions to deliberately and inappropriately engage in SAT if they consider that the only rationale for engaging in SAT development was to avoid JCA. HTDs are unlikely to take the risk to engage in SAT development just to avoid the JCA process, given the expected impact of the inappropriate use of SAT. Additionally, this decision should be endorsed by regulators. Though they may be perceived as less strict than HTA on this matter, they are careful in approving SAT. Since the majority of SAT-supported medicinal products receive positive reimbursement recommendations from HTA, this indicates a fair alignment between HTAs and regulators. Therefore, exempting SAT from JCA is unlikely to encourage more SAT adoption for development.

One may consider that SAT-supported medicinal products should undergo JCA, and the potential downstream effect on market access of a JCA report concluding to a very low degree of certainty may prompt HTDs to evaluate carefully whether to proceed with an SAT development programme as a last resort. The obligation to undergo JCA with a likely low-certainty outcome may reduce the number of SAT-supported products. However, it is unlikely for several reasons. The decision to engage in SAT is primarily made at the regulatory bodies’ level, such as the Food and Drug Administration (FDA) and EMA. Once the decision is made, experience shows that HTA carries little weight in this decision, especially since it is predominantly an EU process and all cost-effectiveness analysis-driven HTA bodies accept unanchored ITC. In the United States, the largest pharmaceutical market, HTA has minimal impact. Furthermore, the US Institute for Clinical and Economic Review (ICER), like other cost-effectiveness analysis-focused organisations, accepts unanchored indirect evidence.

It should be highlighted that if the JCA targets SAT and contributes to the restriction of access to SAT-supported products, it would counteract its primary strategic objective: expanding access to innovative medicinal products in the EU. This action will be monitored by the European Parliament and deemed unacceptable.

Furthermore, the actual influence of JCA on MS HTA outcomes remains unproven. Other impacts of JCA outcomes, which go beyond access and reimbursement, e.g., impact on pricing negotiations, cannot be excluded. However, these are currently unknown and can only be speculative. Mandating all SAT products to undergo JCA and subsequently receive poor assessment conclusions is unlikely to gain support from EU citizens or the European Parliament. On the other hand, if SAT-supported products are exempt from the assessment on the European level, they will still be assessed on the national level, according to current standards and with the current level of acceptability of such treatments.

Finally, the exclusion of SAT-supported products from the JCA due to potential challenges in relative effectiveness assessment might raise a question about whether to exclude RCT-supported products with multiple PICOs, which could lack comparative data to address them. The multiplicity of PICOs is one of the key challenges expected in JCA, independent of the type of study conducted [32]. However, there is a fundamental difference between products with an RCT and SAT. Because of the presence of the control arm in the RCT, it will be possible to address the majority of PICOs resulting from different comparators, either directly or through anchored indirect comparison. In the case of SAT, none of the defined PICOs can be fully addressed for the purpose of comparative relative effectiveness assessment, as only unanchored indirect comparisons are feasible—approaches that are not appropriate for joint relative effectiveness assessment under the Regulation on HTA. It is acknowledged that single-arm trial data may legitimately be included in a JCA report as contextual or supportive information, particularly in rare disease contexts or where no suitable comparator exists, in line with Section 4.3 of the Guidance on Validity of Clinical Studies. The present analysis does not contest the admissibility of SAT-derived data as contextual information; rather, our central research question concerns whether SAT data, as the primary evidentiary basis, can meaningfully support the comparative relative effectiveness assessment that constitutes the JCA’s core statutory output. On that narrower and policy-relevant question, our simulation indicates that 94% of SAT-supported products would not yield a meaningful comparative effectiveness conclusion under the JCA framework as currently designed. It should further be noted that baseline comparisons and comparisons with natural disease progression are methodologically distinct from unanchored indirect treatment comparisons in a strict sense; their potential role as contextual evidence in rare disease contexts is acknowledged, though their acceptability as a basis for comparative effectiveness conclusions under the JCA’s specific harmonised framework remains to be established through practice.

### 4.3. Study Limitations

Choosing France as the reference country may introduce some limitations to this study. However, since HAS reviews all products that have been granted marketing authorisation and for which a submission occurs [24], a set of products identified as having an SAT as the key evidence is likely one of the broadest obtainable through the HTA database analysis. This makes France a reasonable reference for a comprehensive review of SAT-driven submissions in Europe. However, it should be acknowledged as a limitation that the HAS and G-BA databases differ in structural scope and assessment timing: HAS evaluates a broader product range (including additional indications and, in some cases, biosimilars or generics) and frequently assesses products after market entry, whereas AMNOG focuses on early benefit assessments for new active substances at launch. Consequently, using the number of French HAS assessments (96 products) as a universal denominator across all countries introduces a structural non-comparability that affects the cross-country descriptive analysis. This limitation is recognised here explicitly; the cross-country comparison should be interpreted as contextual and descriptive rather than as a basis for causal inference. Two implications follow directly from this design choice and are made explicit here. First, by construction, any SAT-supported product reviewed by G-BA, AOTMiT, or AEMPS but not reviewed by HAS during the study period is excluded from the analysis; the French set serves as the entry point for cross-country tracing, and the magnitude of any such excluded set cannot be quantified from the data at hand. Second, because France serves as the reference country, its count (96 products) constitutes a structural upper bound on the count of products examined in any other country in this study; other countries can, at most, equal but cannot exceed the French count. The numerical gap between France and Germany (96 vs. 65 products), as well as the smaller counts in Poland (40) and Spain (46), is therefore primarily a methodological consequence of the sampling frame, compounded by the structural HTA scope differences noted above, rather than a substantive empirical finding about the relative HTA activity of these countries. Importantly, the country-specific acceptability rates we report (85% France, 94% Germany, 90% Poland, 76% Spain) are computed against country-specific denominators and remain interpretable independently of the cross-country denominator issue. Reassuringly, these country-level findings align closely with previously published independent analyses using different methodologies and sampling frames, including the IQVIA HTA Uncovered analysis and the 2022 ISPOR analysis of SAT-supported products in France and Germany—an empirical convergence supporting the interpretation that the France-as-reference design choice has minimal influence on the validity of the country-specific findings. The central finding of this study—the JCA simulation result showing that 94% of SAT-based products would not qualify for meaningful comparative effectiveness assessment—is derived from the French data alone and is not affected by this cross-country denominator issue.

Due to differences in reimbursement systems across the analysed markets, this study adopted country-specific definitions of acceptability, which complicates the direct comparison of findings. In France, acceptability depends on the SMR rating; products receive acceptance if assigned any rating other than “insufficient.” The SMR assessment evaluates submitted evidence within the disease context. The ASMR further informs pricing by quantifying the added clinical benefit compared to existing alternatives, but does not directly determine acceptability. In Germany, all medicinal products receive reimbursement upon marketing authorization, so HTA outcomes do not affect reimbursement. After assessing the added benefit, pharmaceutical companies must negotiate prices with the National Association of Statutory Health Insurance Funds (GKV), which depends on the G-BA’s assessment [33,34]. An unfavourable assessment could result in an unviable price and prevent the product’s launch in Germany or its withdrawal after HTA or after pricing negotiation. Therefore, market availability can be considered a proxy for the HTA reimbursement decision. In Spain and Poland, the national HTA recommendation is a key evidence-based factor, which allows for the assessment of SAT acceptability. A reimbursement decision following the recommendation is highly driven by non-evidence-based factors like budgetary constraints, delayed adoption due to lengthy negotiations or slow regional dissemination. Therefore, the rate of positive HTA recommendations is the optimum measure of the acceptability in such countries.

Using the JCA degree of certainty as a proxy for acceptability may be considered excessive. However, because MS HTA bodies have to provide due consideration to the JCA report, it may, in these specific cases, be considered as a potential influencer of the HTA outcomes.

JCA may stimulate an increase in indirect evidence generation, which was not assumed for the analysed cases. However, such evidence will always be unanchored. Therefore, according to JCA guidance documents, it will systematically lead to the lowest level of certainty of relative effectiveness. This will rather be a disincentive to submit such evidence.

It remains to be seen if JCA will enhance the number of direct comparative evidence submissions and reduce the number of SAT-supported products. It is very unlikely, as so far, regulatory agencies continue to accept SAT, and obviously, the reimbursement rate and availability on the market are high for such products.

## 5. Conclusions

The proportion of SAT-supported medicinal products enjoy a very high availability, up to 90% in our study countries’ scope. This happens despite a strong requirement for direct comparative evidence. Most products developed with SAT will not be eligible for JCA review, assuming they are filed with current evidence. Even if more products are reviewed by the JCA subgroup for a limited number of PICOs because of HTDs perform more ITC, they will still receive the lowest degree of certainty ratings. This study and the discussion bring some useful learning points:

1. Contextualisation and deliberative processes are critical for SAT and, by essence, overlooked in JCA.
2. If JCA reports are systematically assigned the lowest degree of certainty under the Guidance on Validity for SAT-supported products and this outcome negatively impacts availability at the MS level, it will contradict the primary strategic objective of EU-HTA regulation.
3. If JCA reports are systematically assigned the lowest degree of certainty for SAT-supported products and this outcome does not affect the decision of MS to grant a high rate of availability, this would evidence the low impact of JCA and the redundancy of that process.
4. The JCA process is unlikely to impact the decision of HTDs to engage in SAT development.
5. Exempting SAT from JCA is unlikely to increase the number of SAT-supported medicinal products.

When no JCA report is issued because an SAT is deemed not assessable, MSs may still consider factors such as orphan drug designation, unmet medical need, disease severity, or the absence of treatment alternatives when granting an added benefit or reimbursement. In these situations, the national HTA process could proceed much as it did prior to the introduction of the EU HTA, although the exact impact remains uncertain.

Therefore, it is questionable whether SAT-based products should be considered for JCA, as they may not effectively inform MS HTAs.

## Figures and Tables

**Table 1:** Assessment of the assessability and acceptability of products with SAT under different HTA frameworks.

HTA Framework	Number of Products with SAT	Number of Products Assessed	Number of Products Accepted ^1^	Acceptability Rate
France	96	96	82	85%
Germany	96	65	61	94%
Poland	96	40	36	90%
Spain	96	46	35	76%
JCA	96	6 ^2^	0 ^3^	0%

^1^ Products with an SAT accepted for reimbursement at the HTA stage, at least in one subgroup of patients. The exception is Germany, where all products are accepted by default and can be withdrawn from the market by the manufacturer due to HTA-driven pricing, which is not acceptable for the company. Therefore, in Germany, the acceptance refers to products remaining available on the market after the AMNOG process. ^2^ Anticipated number of assessable products with the availability of data for MAIC or non-adjusted ITC against external control, based on the information included in the French HTA reports. ^3^ Anticipated number of acceptable products based on the degree of certainty of the relative effectiveness assessment.

## Data Availability

No new data were generated during the study.

## Abbreviations

The following abbreviations are used in this manuscript:

AEMPS	Spanish Agency of Medicines and Medical Devices (Agencia Española de Medicamentos y Productos Sanitarios)
AMNOG	German Act on the Reform of the Market for Medicinal Products (Arzneimittelmarktneuordnungsgesetz)
ASMR	Improvement in the actual benefit
ATMP	Advanced therapy medicinal products
EMA	European Medicines Agency
EU	European Union
G-BA	German Federal Joint Committee (Gemeinsamer Bundesausschuss)
HTA	Health Technology Assessment
HTDs	Health Technology Developers
ITC	Indirect treatment comparison
JCA	Joint Clinical Assessment
MAIC	Matching-adjusted indirect comparison
MS	Member States
PICO	Population, Intervention, Comparator, Outcomes
RCT	Randomised control trial
RE	Relative effectiveness
RoB	Risk of Bias
SATs	Single-arm trials
SMR	Rating of actual benefit
TLI	Drug technologies with a high level of innovation
TLK	Drug technologies with high clinical value

## References

[1] Commission Implementing Regulation (EU) 2024/1381 of 23 May 2024 Laying Down, Pursuant to Regulation (EU) 2021/2282 on Health Technology Assessment, Procedural Rules for the Interaction During, Exchange of Information on, and Participation in, the Preparation and Update of Joint Clinical Assessments of Medicinal Products for Human Use at Union Level, as Well as Templates for Those Joint Clinical Assessments. https://eur-lex.europa.eu/legal-content/EN/TXT/?uri=OJ:L_202401381.

[2] Regulation (EU) 2021/2282 of the European Parliament and of the Council of 15 December 2021 on Health Technology Assessment and Amending Directive 2011/24/EU (Text with EEA Relevance). https://eur-lex.europa.eu/eli/reg/2021/2282.

[3] Joint Clinical Assessments. https://health.ec.europa.eu/health-technology-assessment/implementation-regulation-health-technology-assessment/joint-clinical-assessments_en.

[4] Gentilini A., Parvanova I. (2024). Managing experts’ conflicts of interest in the EU Joint Clinical Assessment. BMJ Open.

[5] Guidance on Outcomes for Joint Clinical Assessments. https://health.ec.europa.eu/document/download/a70a62c7-325c-401e-ba42-66174b656ab8_en?filename=hta_outcomes_jca_guidance_en.pdf.

[6] Implementation Rolling Plan. https://health.ec.europa.eu/document/download/397b2a2e-1793-48fd-b9f5-7b8f0b05c7dd_en?filename=hta_htar_rolling-plan_en.pdf.

[7] Methodological Guideline for Quantitative Evidence Synthesis: Direct and Indirect Comparisons. https://health.ec.europa.eu/document/download/4ec8288e-6d15-49c5-a490-d8ad7748578f_en?filename=hta_methodological-guideline_direct-indirect-comparisons_en.pdf&prefLang=el#:~:text=The%20objective%20of%20this%20document%20is%20to%20describe,comparisons%2C%2.

[8] Guidance on Validity of Clinical Studies. https://health.ec.europa.eu/document/download/9f9dbfe4-078b-4959-9a07-df9167258772_en?filename=hta_clinical-studies-validity_guidance_en.pdf.

[9] Wang M., Ma H., Shi Y., Ni H., Qin C., Ji C. (2025). Single-arm clinical trials: Design, ethics, principles. BMJ Support. Palliat. Care.

[10] Single-Arm Trials as Pivotal Evidence for the Authorisation of Medicines in the EU. https://www.ema.europa.eu/en/news/single-arm-trials-pivotal-evidence-authorisation-medicines-eu.

[11] Pharmaceutical Prices (POLI) Database. https://poli.globaldata.com/Home/PricingAndReimbursement.

[12] Download Medicine Data. Orphan Designations. https://www.ema.europa.eu/en/medicines/download-medicine-data#orphan-designations-69050.

[13] Download Medicine Data. https://www.ema.europa.eu/en/medicines/download-medicine-data.

[14] Benefit Assessment of Medicinal Products with New Active Ingredients According to Section 35a SGB V Cerliponase Alfa (Reassessment After the Deadline (Type 2 Neuronal Ceroid Lipofuscinosis)). https://www.g-ba.de/downloads/40-1465-9111/2022-12-15_AM-RL-XII_Cerliponase-Alfa_D-849_TrG_EN.pdf.

[15] Benefit Assessment of Medicinal Products with New Active Ingredients According to Section 35a SGB V Cemiplimab (New Therapeutic Indication: Basal Cell Carcinoma, Locally Advanced or Metastatic). https://www.g-ba.de/downloads/40-1465-8181/2022-01-20_AM-RL-XII_Cemiplimab_D-706_TrG_EN.pdf.

[16] Beschlüsse über die Nutzenbewertung von Arzneimitteln mit neuen Wirkstoffen nach § 35a SGB V—Sofosbuvir. https://www.g-ba.de/downloads/40-268-2899/2014-07-17_AM-RL-XII_Sofosbuvir_2014-02-01-D-091_TrG.pdf.

[17] Novartis Withdraws Tabrecta in Germany. https://www.navlindaily.com/article/18714/novartis-withdraws-tabrecta-in-germany.

[18] Bluebird to Withdraw Gene Therapy from Germany After Dispute over Price. https://www.biopharmadive.com/news/bluebird-withdraw-zynteglo-germany-price/598689/.

[19] Gavreto (Pralsetinib)—Withdrawal of the Marketing Authorisation in the European Union. https://www.ema.europa.eu/en/documents/public-statement/public-statement-gavreto-withdrawal-marketing-authorisation-european-union_en.pdf.

[20] Assessment of Reimbursement Applications. https://www.aotm.gov.pl/en/medicines/assessment-of-reimbursement-applications/.

[21] Fundusz Medyczny D Subfundusz Terapeutyczno_Innowacyjny (STI). https://www.gov.pl/web/zdrowie/d-subfundusz-terapeutyczno-innowacyjny-sti.

[22] Macabeo B., Rotrou T., Millier A., François C., Laramée P. (2024). The Acceptance of Indirect Treatment Comparison Methods in Oncology by Health Technology Assessment Agencies in England, France, Germany, Italy, and Spain. PharmacoEcon. Open.

[23] Rémuzat C., Toumi M., Falissard B. (2013). New drug regulations in France: What are the impacts on market access? Part 2—Impacts on market access and impacts for the pharmaceutical industry. J. Mark. Access Health Policy.

[24] Transparency Committee Doctrine. https://www.has-sante.fr/upload/docs/application/pdf/2019-07/doctrine_de_la_commission_de_la_transparence_-_version_anglaise.pdf.

[25] Ivandic V. (2014). Requirements for benefit assessment in Germany and England—Overview and comparison. Health Econ. Rev..

[26] Kranz P., McGauran N., Ünal C., Kaiser T. (2024). Results of health technology assessments of orphan drugs in Germany-lack of added benefit, evidence gaps, and persisting unmet medical needs. Int. J. Technol. Assess. Health Care.

[27] Schulz S., Passon A.M., Perleth M., Kulig M., Paschke N., Matthias K. (2020). The Evaluation of Orphan Drugs by the German Joint Federal Committee—An Eight-Year Review. Dtsch. Arztebl. Int..

[28] HTA Uncovered. https://www.iqvia.com/-/media/library/white-papers/hta-uncovered-july-2016.pdf.

[29] Health Technology Assessments of Single-Arm Clinical Trials in Germany and France. https://www.ispor.org/docs/default-source/euro2022/poster-ispor-eu-2022final-pdf.pdf?sfvrsn=db81698c_0.

[30] Use of Single Arms Studies for Health Technology Assessment in Portugal. https://www.ispor.org/docs/default-source/euro2022/posterhta-pdf.pdf?sfvrsn=3628267b_0.

[31] Practical Guideline for Quantitative Evidence Synthesis: Direct and Indirect Comparisons. https://health.ec.europa.eu/document/download/1f6b8a70-5ce0-404e-9066-120dc9a8df75_en?filename=hta_practical-guideline_direct-and-indirect-comparisons_en.pdf.

[32] Schuster V. (2024). EU HTA Regulation and Joint Clinical Assessment—Threat or Opportunity?. J. Mark. Access Health Policy.

[33] Benefit Assessment of Medicinal Products. https://www.g-ba.de/english/benefitassessment/.

[34] Blümel M., Spranger A., Achstetter K., Maresso A., Busse R. (2020). Germany: Health System Review. Health Syst. Transit..

